# The Boarding School Nuisance

**Published:** 1856-08

**Authors:** 


*The Boarding School Nuisance.*—While our Sanitary Police is engaged in inspecting emigrant boarding houses, the tenant houses of the poor, and in ferreting out the causes of disease in the alleys and unventilated courts of cities, equally fruitful sources of ill-health exist among our higher classes, producing evils as serious and as lasting.

A few weeks ago we were called to see a young girl suffering from general debility, neuralgic pains, vertigo and headache. She had just returned from a boarding-school in a neighboring city, where she had spent only a month before her health, previously good, failed. On inquiry we found the routine of the school to be as follows, and to be certain of the correctness of her account we have made inquiries of others familiar with its management.

The pupils rise at five in the morning. They study from five to seven o'clock. From seven to eight o'clock they have breakfast. From eight in the morning to two, P. M., is spent in the schoolroom—a period of six hours. At two they have dinner; and from three to five are allowed to walk or take other exercise. From five to six they study; at six have tea, and then study from seven to nine, when they are sent to bed.

Their diet is light and unsubstantial, and their appetites under such a regimen, are as feeble as the diet.

Now here the day of a young, growing, spirited school-girl is divided into periods of seven hours for sleep, three for meals, two for exercise, and twelve for study. Every person under full adult age needs eight or nine hours' sleep, and in order that that sleep should be healthful and refreshing, they require at least six hours of recreation and active exercise. The time for meals is sufficiently ample in the instance here mentioned, but to allow only two hours for exercise, and that in the afternoon when heat and fatigue dispose them to rest, is positively murderous. And twelve hours study per day is at least six hours too much for any young person.

A child in full, vigorous health, will acquire more knowledge in six hours daily, than in twelve; for full health and mental vigor is incompatible with the discipline we have described.

This system of education takes young, robust, romping girls, and transforms them to slow, languid, pale, worthless women. To acquire skill on the piano, a little bad French, and a namby-pamby knowledge of a few of the "English branches," they sacrifice health, energy, all capacity for the duties of womanhood, and not unfrequently life itself.

Our prescription in the case on which these remarks are founded, was, of course, a simple one — to stay at home and abjure boarding-schools.

